# Disasters in Spain from 1950 - 2020: Impact on Public Health

**DOI:** 10.1017/S1049023X23000225

**Published:** 2023-04

**Authors:** Pedro Arcos González, Nel Suárez Ruiz, Rafael Castro Delgado, José Antonio Cernuda Martínez

**Affiliations:** 1. Unit for Research in Emergency and Disaster, Department of Medicine, University of Oviedo, Oviedo, Spain; 2. Health Service of the Principality of Asturias (SAMU-Asturias), Oviedo, Spain; 3. Institute for Health Research of the Principality of Asturias, Oviedo, Spain

**Keywords:** disasters, mortality, public health

## Abstract

**Objective:**

The aim of this study was to establish the frequency and profile of disasters and to analyze trends in disasters and their impact on Spanish public health.

**Methods:**

Retrospective observational study of disasters that occurred in Spain from 1950 through 2020 was conducted. The variables studied for each episode were number of people affected, number of injured/sick, and number of deaths. Absolute and relative frequencies, population rates, mean, median, standard error of the mean (SEM), and 95% confidence intervals (CI) were used, and trend analysis was performed using exponential smoothing and linear regression.

**Results:**

A total of 491 disasters were identified in Spain. Of these, 255 (51.9%) were natural disasters, 224 (45.7%) technological disasters, and 12 (2.4%) man-made disasters. The average number of disasters per year was 7.01 (95% CI, 5.99-9.34). These disasters affected a total of 820,489 people, with an average of 3,491 people (SEM = 2.18) per episode. There was a significant increase (P <.001) in the total frequency of disasters in Spain during the period studied.

**Conclusions:**

Spain has a disaster profile of mixed type, combining natural with technological disasters. From 1950 through 2020, there was a significant increase in the number of disasters, with an overall profile similar to that of Europe, with climatological disasters being the most frequent type.

## Background

Disasters are complex situations in which the consequences of an event are beyond the capability of an affected jurisdiction to respond effectively. A disaster is defined as: “a serious disruption of the functioning of a community or a society causing wide-spread human, material, economic, or environmental losses that exceed the ability of the affected community or society to cope using its own resources, thus necessitating a request to national or international level for external assistance.”^1,2^ They can be of two types: natural (hydrometeorological, geological, or biological) or human-made disasters (unintentional or deliberate – eg, terrorist attacks including terrorism, chemical spills, wildfires, engineering failures, or civil conflicts).^3–6^

Disasters are one of the major threats facing society’s health nowadays. In recent decades, the number of disasters has increased and led to many deaths, injuries, diseases, and disabilities.^7,8^ Moreover, they have an increasing, large-scale impact on the public health of affected populations.^9,10^ From 2000 through 2019, a total of 7,348 major disaster events were recorded world-wide, affecting 4.2 billion people, killing 1.23 million, and causing approximately US$2.97 trillion worth of damage. This increase in disaster frequency and impact over the last 20 years is unprecedented.^11^ Disasters related to climatological hazards,^12^ complex humanitarian emergencies with population displacement,^13^ and epidemic emergencies are the types of disasters that are likely to pose the greatest challenges throughout the world in the coming decades.^14^ In Spain, as in other European countries, the frequency and impacts of climatological disasters, especially extreme weather events like floods, droughts, and wildfires, will be especially intense due to its geographical position.^15,16^

The trends in the frequency of disasters and their impacts on mortality and morbidity in Spain have been studied up to 2012, but no such analysis has been conducted for the period between 2012 and the present.^17^ Therefore, it seems necessary to extend the study period to analyze the changes that have occurred in the last decade, as well as to improve the accuracy of the analysis of the epidemiological trends of the phenomena and their consequences over the whole period. The aim of this study was to establish the frequency and profile of disasters that have caused personal injuries or deaths in Spain and to analyze their trends from 1950 through 2020.

## Material and Method

Retrospective observational study of disasters occurring in Spain from January 1, 1950 through December 31, 2020 was conducted using the Centre for Research on the Epidemiology of Disasters (CRED; Brussels, Belgium) definition of disaster: an event was considered to constitute a disaster if it resulted in ten or more deaths, 50 or more injuries, and/or 50 or more people affected.^18^ For each disaster episode, the following variables were studied: number of people affected, number of injured/sick people, and number of deaths.

The data sources used for the identification of the episodes were of primary level: disaster databases and information from organizations of accredited international prestige, such as the CRED, Red Cross-Red Crescent (IFRC; Geneva, Switzerland), World Health Organization (WHO; Geneva, Switzerland), United Nations Office for the Coordination of Humanitarian Affairs (OCHA; New York, USA), as well as Spanish agencies related to subgroups or specific types of disasters as the Directorate General of Civil Protection (Madrid, Spain), Ministry of Interior (Madrid, Spain), Ministry of the Environment (Madrid, Spain), and Ministry of Health (Madrid, Spain); and also of secondary level (mainly national and regional newspapers, which are sources of information that provide information on specific episodes and their impact on morbidity and mortality). The first-level sources generally offer global aggregate data but insufficient information on specific episodes.

Absolute and relative frequencies as well as population rates were used in the statistical analysis. Figures for population at risk were provided by the Spanish Institute of Statistics (INE; Madrid, Spain) in the year in which each episode occurred.^19^ The mean, median, standard error of the mean (SEM), and 95% confidence intervals (CI) were used. Variables were analyzed using the Shapiro-Wilk test to study the normality in time series, and trend analysis was performed using exponential smoothing and linear regression. Data processing and statistical analysis procedures were performed with SPSS Statistics v25 (IBM; Armonk, New York USA).

## Results

From 1950 through 2020, a total of 491 disasters were identified in Spain. Of these, 255 (51.9%) were natural disasters, 224 (45.7%) were technological disasters, and 12 (2.4%) were man-made disasters. The average number of disasters per year was 7.01 (95% CI, 5.99-9.34). Table 1 shows the values of the basic statistical parameters of the distributions of the frequency of episodes, casualties, injured, and death rates, as well as those of the analysis of the normality of the variables.

**Table 1.** Values of the Basic Statistical Parameters of the Distributions of the Frequency of Episodes, Casualties, Injured, and Death Rates

	Number of Episodes (n)	Mortality Rate x 10^8^	Morbidity Rate x 10^8^	Injured Rate x 10^8^
Absolute Frequency (n)	491	64	63	61
Mean by Year	7.67	2859.48	66789.62	33160.56
Standard Error of Mean	0.84	2464.66	64618.57	22003.36
Median	5	180.86	694.92	177.92
Range	1 to 29	10.43 to 158082.37	0 to 4072557.6	0 to 1330317.3
Confidence Interval 95%	5.99 to 9.34	0 to 7784.71	0 to 195960.3	0 to 77173.85
Skewness	1.63	7.99	7.93	7.43
Kurtosis	2.17	63.91	62.96	56.66

The 491 disasters that occurred in Spain during the study period affected a total of 820,489 people. There were people affected in 235 (47.8%) episodes, with an average of 3,491 (SEM = 2.18) affected people per episode. Of the total number of people affected, 761,281 (92.8%) were affected by a natural disaster, 59,208 (7.2%) by a technological one, and there were no people affected by man-made disasters.

In the same period (1950-2020), 218 (44.4%) of the disasters that occurred resulted in a total of 1,981,184 wounded, injured, or sick people, with an average of 4,288 per episode (SEM = 4.17). Of the total number of wounded, injured, or sick, 1,950,048 (98.4%) were due to a natural disaster, 28,528 (1.3%) to a technological disaster, and 2,608 (0.1%) to a man-made disaster.

In the same period, 228 disasters (46.4%) of the 491 that occurred caused the death of a total of 83,932 people in Spain, with an average of 171 casualties per episode (SEM = 152.7). Of these casualties, 5,685 (6.7%) occurred in a technological disaster, 77,934 (92.9%) in a natural disaster, and 312 (0.4%) in a man-made disaster.

**Figure 1.**
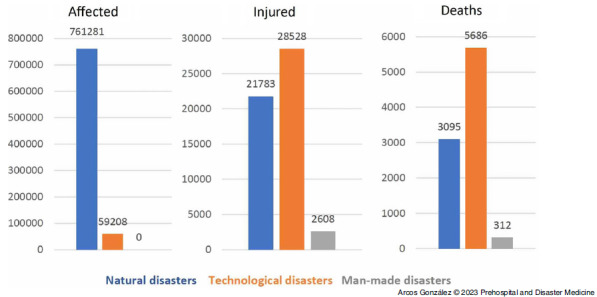
Frequency of Affected and Injured People, as well as Casualties, by Type of Disaster from 1950 through 2020 in Spain.

**Figure 2.**
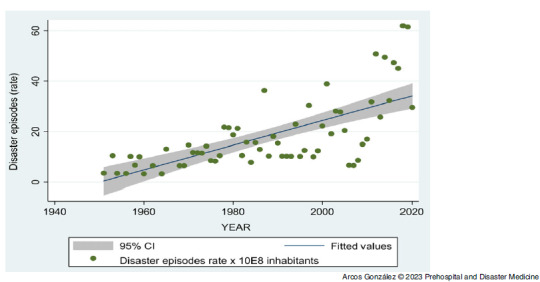
Time-Trend Analysis of the Annual Disaster Rate from 1950 through 2020 in Spain.

**Figure 3.**
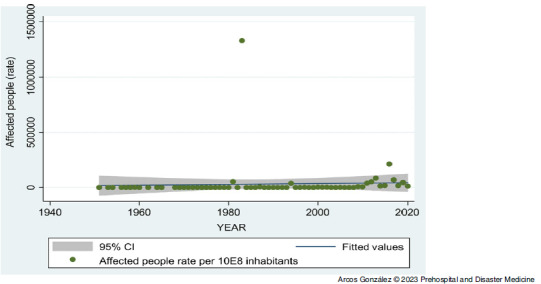
Time Series Analysis of the Rate of People Affected by Disasters from 1950 through 2020 in Spain.

Figure 1 shows the frequency of affected and injured people as well as casualties by type of disaster, excluding the figures for the coronavirus disease 2019 (COVID-19) pandemic in 2020. Figure 2 shows the time-trend analysis of the annual disaster rate from 1950 through 2020. As can be seen, there has been a significant increase (P <.001) in the frequency of disasters in Spain during the period studied. Figure 3 shows the time series analysis of the rate of people affected by disasters. The high numbers of affected people in the years 1983 and 2016 correspond to intense episodes of flooding in different parts of Spain (Basque Country in the north and Eastern Regions of the country, respectively). Although there has been an increase in the number of people affected by disasters in Spain in the last decade, this trend has not been statistically significant.

**Figure 4.**
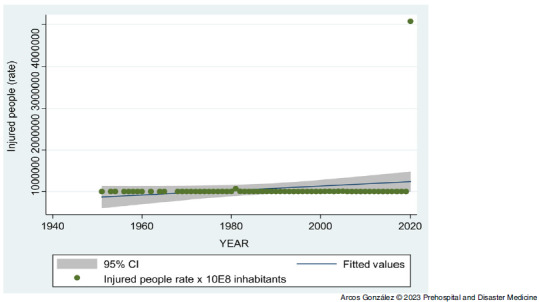
Time Series Analysis of the Morbidity Rate (Wounded, Injured, or Sick) due to Disasters from 1950 through 2020 in Spain.

**Figure 5.**
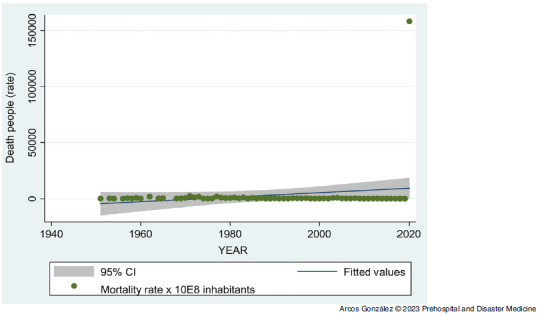
Time Series Analysis of the Disaster Mortality Rate from 1950 through 2020 in Spain.

Figure 4 shows the time series analysis of the morbidity rate (wounded, injured, or sick) due to the disasters. The peak of casualties in the year 2020 (1,928,356) corresponds mostly (1,928,265; 99.9%) to the COVID-19 pandemic cases. Despite this high value, the series as a whole has not shown a statistically significant increase in the number of people injured or sickened by disasters in Spain. Figure 5 shows the time series analysis of the disaster mortality rate. Again, the strong increase in deaths (74,839) in 2020 corresponds mostly (74,826, 99.9%) to deaths due to the COVID-19 pandemic. Despite that increase, for the whole period studied, no statistically significant increase in mortality due to disasters has been found.

## Discussion

The aim of this study was to study the frequency, profile, and trends of disasters in Spain from 1950 through 2020. In this period, the frequency of disasters in Spain has increased, and this increase has been more pronounced in the last 40 years. However, the smaller amount of information available in the previous decades could cause an under-estimation of the data in that period.^20^

The Spanish disaster profile is similar in terms of the type of disaster to the global profile, since the most prevalent type has been the meteorological/climatological disasters, followed by geophysical and biological types. The African continent constitutes an exception to this, since their biological disaster episodes outnumber geophysical ones.^9^

Since the end of the 20th century, the frequency and severity of disasters have increased throughout the world.^21–23^ Regardless of their natural or technological origin, this increase has been quantitative and qualitative.^24^ Different studies link the increase in the number of disasters to the industrialization processes of countries, urbanization and environmental degradation, and climate change.^23,25–27^ These risk elements generate synergies that increase both the frequency and severity of disasters, as well as the vulnerability of populations and the degree to which they are affected.^27^

World-wide, most natural disasters now have a climatological origin, in particular floods, storms, droughts, and wildfires. Moreover, disasters of the climatological type have been consistently increasing in frequency over the last decades, from 76% to 83%.^10,11^ In contrast, geophysical disasters have remained stable in frequency. In Europe, extreme temperature events caused the highest number of human fatalities, while flooding and storms were the costliest hazards. There has also been an increase in communicable disease outbreaks since the 1960s.^21,23^

These global patterns and trends are also replicated in Spain. More specifically, in the period from 1950 through 2012, most disasters in Spain (57%) were technological, followed by natural (39%) and human-induced (4%).^17^ Over the course of the interval from 1950 through 2020 analyzed in this study, it can be noted that from 2012 through 2020, natural disasters have increased while technological and human-induced disasters have decreased.

The results of the dispersion parameters of the distributions of those affected, injured, or ill and of casualties per disaster in Spain reveals a great variability in the magnitude of the episodes in terms of their impact on mortality and morbidity.

The increase in the frequency of disaster episodes in Spain, together with the fact that that increase has not translated into significant increases in the number of people affected, injured, or killed, could suggest an improvement in prevention, preparedness, mitigation, and response capacities over the last 70 years. Although several European disaster policies have already been adopted or initiated in Spain, more effort is needed to implement an integrated risk management system for all hazards. The difficulties that were experienced during the study in finding relevant and integrated information on disasters in Spain suggest that further efforts must be made to compile and organize disaster information, which is currently scattered over various sources, because disaster risk reduction and management rely on the availability of solid evidence.

## Limitations

A common limitation when studying the epidemiology of disasters, and which also affects the case of this study, is the absence of a “quantitative” definition of the phenomenon. Although there are definitions of what a disaster is, the absence of common quantitative criteria on the impacts (ie, minimum numbers of dead or affected) to be considered as inclusion criteria limits the comparability of the databases and the comparability between countries.

## Conclusions

Spain has a disaster profile of a mixed type, combining natural and technological disasters, but during the last decade, the natural disasters have increased while technological and human-induced disasters have decreased. These types of disasters differ greatly in magnitude and impact in terms of mortality and morbidity.

In the period from 1950 through 2020, there has been a significant increase in the number of disaster episodes in the country, and the profile in terms of number and type of disaster has been like the rest of Europe where climatologically disasters have been predominant.

Natural disasters are the type of phenomenon that have caused the most deaths, injuries, and casualties in Spain in recent decades, while floods in particular affected the greatest number of people.

In the last decade, there has been an increase in the number of people affected by disasters, but this growing trend has not been statistically significant for the period. Likewise, there has been no significant increase in the number of people injured, wounded, or ill due to disasters in Spain, nor in the number of deaths due to disasters, except for the increase in 2020 corresponding to the COVID-19 pandemic.
